# Tribute of Respect to the Memory of Dr. Wm. H. Butler

**Published:** 1864-03

**Authors:** 


### TRIBUTE OF RESPECT TO THE MEMORY OF DR. WM. H. BUTLER.

At a meeting of the Physicians of Buffalo, held February 12th, 1864, at the rooms of the Buffalo Medical Association, on the occasion of the death of Dr. William H. Butler, it was, on motion, resolved: That as a token of respect for the memory of the deceased, we will attend his funeral in a body, and wear the usual badge of mourning, and that a portion of our members in accordance with the wishes of his family, will act as bearers of his remains. A committee was also appointed to draft suitable resolutions,

Dr. White spoke briefly, eulogizing the deceased as one who had taken up the study of his profession out of a pure love for it. A young man of more than ordinary talent, he pursued the practice of medicine with zeal, and contributed to the *Medical Journal* during his brief career, a number of valuable articles upon subjects connected with his profession.

Dr. Peters said he had known Dr. Butler for several years, both in civil life and in the military service. He would leave others who knew him better so speak of his life here, but wished to testify his respect for him while in service. Dr. Butler came out in the fall of 1861, as Assistant Surgeon of the Sixteenth Michigan Volunteers. The regiment on their entry into service suffered a good deal from sickness, the Surgeon being himself sick, and Dr. Butler had a good deal of severe labor thrown on his hands, more than he was physically capable of doing. When the army moved to the Peninsula, Dr. Butler was sick in Alexandria from exposure in the field, and was unable to join his regiment. He was advised by all who knew him to resign, but refused to do so, and as soon as able joined the army in the field. Here he remained until entirely broken down, and was discharged for disability. He was then employed as contract Surgeon in the Army Square Hospital, Washington, and was also appointed Examining Surgeon to the Pension Bureau. On this duty he remained until his death. His name must be added to the long list of those of our profession, who have worn themselves out in the service of our country during the rebellion. He was repeatedly advised that he might prolong his life by seeking another climate and less arduous duties, but always refused to do so, preferring to remain at his post, consciously sacrificing his life to his sense of duty.

The following resolutions were adopted:

Resolved—That in the death of William H. Butler, M. D., the medical profession has lost an earnest, honorable and able representative.

Resolved—That his career in the army, and in the Military Hospital at Washington, affords striking evidence of courage, perseverance and humanity manifested under constant and increasing physical infirmity,

Resolved—That we sympathize most deeply with his bereaved wife and children, and tender to them our heartfelt condolence.

Resolved—That a copy of our proceedings, and these resolutions, be sent to his family, and that their publication be requested in the *Buffalo Medical Journal* and the city papers.

LEON F. HARVEY, Sec'y. J. B. SAMO, Chairman.

ARMORY SQUARE HOSPITAL,
*Washington, D. C., February* 6, 1864.

It becomes our painful duty to announce the death of Dr. BUTLER, late Assistant Surgeon in this Hospital. He was thoroughly educated and skillful in his profession. He was beloved by his associates in office, and by all the patients who were so fortunate as to be under his care. His faith being strong in the promises of a gracious Redeemer to the last, his end was peaceful and triumphant. Dr. B. had served his country faithfully from the commencement of the war, and fallen at his post. The Masonic Fraternity, of which he was a member, took charge of his remains and have removed them for interment to Buffalo, N. Y., the place of his former residence. The following are the resolutions adopted by the officers of this institution:

At a meeting of the Officers of Armory Square Hospital, Washington, D. C., held this 6th inst., at the office of the Surgeon in Charge, called upon the occasion of the death of their fellow officer, Act. Ass't Surgeon W. H. BUTLER, who died February 5th, after a lingering illness of several months, from Tubercular Consumption, the following was resolved:

That in this dispensation of an all-wise Providence, we recognize the hand of Him "That doeth all things well," and while we are grieved to part with one endeared to us by so many qualities of the gentleman and officer, we meekly bow, feeling "What is our loss is his gain."

Resolved—As a brother officer, having during the entire war been in the service of his country, in which he has died, and having patriotically left a large practice for it, we part with him with more than ordinary feelings of regret; for by his assiduous attention to his patients; his skillful practice; his gentlemanly and officer like demeanor, we feel in him our profession has lost a noble man a faithful student.

Resolved—That to his bereaved wife and family, we extend our most cordial and heart-felt sympathies, feeling while in this loss they have been bereft of an affectionate and faithful husband and father, there is "One who is a father to the fatherless, and a husband to the widow," and who will not desert them in this their hour of need.

Resolved—That a copy of the above resolutions be forwarded to the family of the deceased, and be published in the *Army Square Hospital Gazette*, *Washington Chronicle*, *Republican*, and *Buffalo Medical Journal*.

